# 

**Published:** 1918-08-24

**Authors:** 


August 24th, 1918.] [Price Twopence

### The Hospital

The Workers' Newspaper of Administrative Medicine and Institutional Life, Administration, National Insurance and Health.

CONTENTS.

Medical Opinion and Organisation ... ... ... 441
The B.M.A. Representative Meeting.
Anopheline Mosquitoes in Britain ... ... 442
Unfavourable Breeding Ground for Malaria.
Papers on Madness ... ... ... ... 447
II. Horrors and Abuses of the Old Madhouses.
The Simulation of Disease ... ... ... ... 449
I. The State and the Malingerer.
Westminster and King's College Hospitals ... 450
British Science During the War ... ... ... 452
Hospital and Institutional News ... ... ... 443
King George's Fund for Sailors—The Forthcoming Hospital Conference—The King and the Disabled—British Doctors Receive Italian Honours—America and the Royal Free Hospital—Lotteries and Italian Hospitals—Staffs, Salaries, and the War Loan—A Welsh Enthusiast—The Vitality of the Voluntary System—The Latest Register of Charities—Better Bread for Wounded Soldiers—The Progress of Army Dentistry—A Cry from Kashmir—A Permanent Stop-gap—Welfare Work in its Infancy—A Brighton Hospital's Jubilee—The *American Red Cross Bulletin*—The Relief of War Distress—Curious Air-raid Effects—War Bonus to Worcester Nurses—Willesden's Municipal Clinic—This Week's Drug Market—To Our Readers.

CONTENTS.

The Editor's Letter-Box ... ... ... ... ... 451
Guest Hospital, Dudley—The Insurance Act and its Administrators—London Hospital Nurses: Miss Lynette Gould's View; Miss McKinley Withdraws her Statements—A Nurses' Charter of Liberty.
The Matrons' and Sisters' Department ... ... 453
The Constitution of the Local Centre.
Round the Hospitals ... ... ... ... ... ... 453
The Queen as Hospital Visitor—1,600 Mentions for Hospital and Nursing Services—How to Double the Hot-water Supply—The First American V.A.D.s—Increased Salaries at Hartshill—Medical Women to Inspect Midwives—17,000 Unregistered Nurses in America.
The Institutional Library ... ... ... ... 459
The Mother and the Child—Painless Childbirth in Twilight Sleep in the East—How to Enlighten our Children—National Feeding of Infants—The Baby—My War Diary—With the Scottish Nurses in Roumania.
The "Women's Ambulance" in a Shell Factory... 461
State Aid for the Working Man's Child ... ... 461
The Public Health ... ... ... ... ... 458
Matrons' and Sisters' Appointments ... ... ... 462
Recent Official Publications ... ... ... ... 462
Institutional Worker ... ... ... ... ... 1

